# Timing of initiation of enzyme replacement therapy after diagnosis of type 1 Gaucher disease: effect on incidence of avascular necrosis

**DOI:** 10.1111/j.1365-2141.2009.07872.x

**Published:** 2009-11

**Authors:** Pramod K Mistry, Patrick Deegan, Ashok Vellodi, J Alexander Cole, Michael Yeh, Neal J Weinreb

**Affiliations:** 1 Department of Pediatrics, Yale University School of MedicineNew Haven, CT, USA; 2 Addenbrooke’s HospitalCambridge; 3 Great Ormond Street Hospital for Children NHS TrustLondon, UK; 4 Genzyme CorporationCambridge, MA; 5 University Research Foundation for Lysosomal Storage Diseases, Inc.Coral Springs, FL, USA

**Keywords:** Gaucher disease, enzyme replacement therapy, avascular necrosis, imiglucerase

## Abstract

Data from the International Collaborative Gaucher Group Gaucher Registry were analysed to assess the relationship between enzyme replacement therapy with imiglucerase (ERT) and incidence of avascular necrosis (AVN) in type 1 Gaucher disease (GD1), and to determine whether the time interval between diagnosis and initiation of ERT influences the incidence rate of AVN. All patients with GD1 enrolled in the Gaucher Registry who received ERT and did not report AVN prior to starting therapy (*n* = 2700) were included. The incidence rate of AVN following initiation of ERT was determined. An incidence rate of AVN of 13·8 per 1000 person-years was observed in patients receiving ERT. Patients who initiated ERT within 2 years of diagnosis had an incidence rate of 8·1 per 1000 person-years; patients who started ERT ≥2 years after diagnosis had an incidence rate of 16·6 per 1000 person-years. The adjusted incidence rate ratio was 0·59 [95% confidence interval (CI) 0·36–0·96, *P* = 0·0343]. Splenectomy was an independent risk factor for AVN (adjusted incidence rate ratio 2·23, 95% CI 1·61–3·08, *P* < 0·0001). In conclusion, the risk of AVN was reduced among patients who initiated ERT within 2 years of diagnosis, compared to initiating treatment ≥2 years after diagnosis. A higher risk of AVN was observed among patients who had previously undergone splenectomy.

Gaucher disease (GD), the most common lysosomal storage disorder, results from defective activity of acid β-glucosidase (EC 3.2.1.45; lysosomal glucocerebrosidase) due to mutations in the *GBA* gene. Enzyme deficiency leads to accumulation of glucocerebroside in the lysosomes of mononuclear phagocytes and a complex multisystemic phenotype (Grabowski *et al*, 2006). The glucocerebroside-engorged macrophages trigger a chronic inflammatory state, immune dysfunction and, occasionally, fibrosis (Cox & Schofield, 1997). The estimated prevalence of GD is 1/57 000 (Meikle *et al*, 1999) to 1/75 000 newborns (Grabowski, 2005) although its prevalence in people of Ashkenazi Jewish ancestry is higher, at *c*. 1 in 500 (Weinreb *et al*, 2008). Type 1 GD (GD1) comprises *c*. 94% of all cases, and it is differentiated from type 2 and type 3 GD by the absence of primary central nervous system involvement (Grabowski *et al*, 2006).

The most prominent sites of pathology in GD1 are the liver, spleen, bone marrow, skeleton and lungs (Charrow *et al*, 2000; Grabowski *et al*, 2006). Clinical and radiological evidence of diverse skeletal involvement occurs in the majority of patients (Charrow *et al*, 2000; Grabowski *et al*, 2006; Weinreb *et al*, 2007). However, skeletal involvement may occur in the absence of significant haematological and visceral abnormalities, underscoring the extreme heterogeneity of GD1 within the same genotype groups and even among affected siblings (Taddei *et al*, 2009).

Type 1 GD is a progressive condition in which irreversible disease sequelae can occur in untreated patients (Kaplan *et al*, 2006; Maaswinkel-Mooij *et al*, 2000; Taddei *et al*, 2009). A major irreversible complication of GD1 is avascular necrosis (AVN), which can lead to joint destruction, the need for joint replacement surgery, and chronic disability. AVN may rarely result in pseudotumors of the bone known as ‘Gaucheromas’ and pathological fractures (Elstein *et al*, 1997). In the natural course of GD1, the occurrence of AVN appears to be unpredictable and its risk factors are not understood.

The standard of care for GD1 is enzyme replacement therapy with imiglucerase (ERT). While enzyme therapy is highly effective in reversing visceral and haematological manifestations and some aspects of skeletal disease, its precise impact on the risk of bone disease, such as AVN, is not known (Andersson *et al*, 2005; Barton *et al*, 1991; Charrow *et al*, 2007; Cox *et al*, 2008; Grabowski *et al*, 2006, 1998; Sims *et al*, 2008; Weinreb *et al*, 2007, 2002; Wenstrup *et al*, 2007). A key unanswered question regarding the management of GD1 concerns the timing of treatment initiation, and whether early initiation of ERT after diagnosis reduces the incidence rate of clinically significant events, such as AVN. The present study used the International Collaborative Gaucher Group (ICGG) Gaucher Registry database to determine whether the rate of AVN following treatment initiation varied according to particular risk factors, including the interval between diagnosis and initiation of enzyme therapy. Our results provide evidence on which clinical decisions regarding the optimal timing to begin ERT can be based.

## Methods

### ICGG Gaucher Registry

The ICGG Gaucher Registry was launched in 1991 to track the clinical, demographic, genetic, biochemical and therapeutic characteristics of patients with GD throughout the world, irrespective of disease severity and treatment status (Charrow *et al*, 2000). The goals of the Registry are to define the clinical spectrum of GD, assess its natural history though longitudinal follow-up and assess the effect of treatment. An independent international group of physicians who are experts in GD provide scientific direction and governance of the Registry, with logistical support from Genzyme Corporation (Cambridge, MA, USA). Since 1991, with Institutional Review Board/Ethics Committee approvals, over 700 physicians from 60 countries have voluntarily submitted de-identified data on over 5000 patients to the Registry.

### Study population

Data included in this study were recorded in the Registry as of July 2007. ERT refers to mannose-terminated glucocerebrosidase, whether human placenta-derived, alglucerase (Ceredase®; Genzyme Corporation) or human recombinant Chinese Hamster Ovary cell-generated, imiglucerase (Cerezyme®; Genzyme Corporation). Alglucerase and imiglucerase have been shown to be therapeutically equivalent in a randomized, two-arm clinical trial (Grabowski *et al*, 1995). Of the patients included in this analysis, 44% initiated therapy with alglucerase and 56% initiated therapy with imiglucerase. Within several years, most of the patients who initiated therapy with alglucerase eventually switched to imiglucerase; only 16% of patients were treated solely with alglucerase during follow-up.

The dataset for this analysis included all GD1 Registry patients who received imiglucerase with the following information: date of GD diagnosis; age at GD diagnosis; dates of first and last assessments in the Registry; and date of initiation of therapy. The diagnosis of GD1 was usually based on the assay of acid β-glucosidase activity and/or genotyping of *GBA*.

The objective of the analysis was to estimate the incidence rate of new onset AVN following treatment initiation. Therefore, patients with AVN reported before initiation of therapy were excluded from this analysis.

### Demographic and clinical characteristics of patients

Patient demographics and clinical manifestations of GD1 were characterized at the time of therapy initiation. Clinical manifestations included whether prior therapeutic splenectomy had been performed, haemoglobin concentration, platelet count, spleen volume, liver volume, bone mineral density and reports of bone pain and/or bone crisis. Criteria for diagnosis of bone crises in the Registry include acute onset of bone pain requiring immobilisation of the affected area and narcotics for the relief of pain, accompanied by one or more of the following: periosteal elevation, elevated white blood cell count, elevated inflammatory markers, fever or debilitation >3 d (Charrow *et al*, 2007). This definition excluded fracture and osteomyelitis. Bone pain was defined as patient-reported pain experienced during the 30-d period preceding the report and attributable to Gaucher disease according to the clinical judgment of the reporting physician.

### Ascertainment of avascular necrosis

Avascular necrosis was typically ascertained from X-ray or magnetic resonance imaging (MRI) results. The recommended radiological guidelines for patients with GD1 include assessment of the hip, femur, pelvis and lumbar spine. The date of onset and site of AVN was recorded on the skeletal assessment case report forms of the Registry. If a patient had AVN reported on more than one date, the patient’s earliest date of AVN onset was identified.

### Person-time of follow-up

Follow-up began on each patient’s date of initiation of therapy in the Registry. Patients were followed until either the date of AVN onset, or for patients who did not develop AVN, the date of their last recorded assessment in the Registry. Each patient was classified according to the duration of time between diagnosis of GD1 and initiation of therapy. The categories were as follows: <1; 1–<2; 2–<5; 5–<10; 10–<20; 20–<30; and 30+ years.

### Data analysis

Descriptive statistics were used to analyse data in this study according to demographics and clinical characteristics of GD1. Proportions were calculated for categorical variables (i.e. gender, genotype, ethnicity). Summary statistics (mean, standard deviation, percentiles) were calculated for continuous measures (i.e. age). Incidence rates were calculated as follows: 
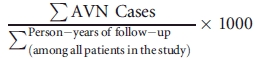


Other candidate risk factors for new onset AVN were collectively examined through construction of a multivariate Poisson regression model. Variables in the model included interval between GD diagnosis and initiation of therapy (<2, 2 years or more), age at initiation of therapy, age at Gaucher diagnosis, gender, year of diagnosis (before 1991, 1991–1999, 2000 or later), splenectomy before initiation of therapy, calendar year of initiation of therapy (before 1995, 1995–1999, 2000 or later), and genotype category (N370S/N370S, N370S/Other, Other/Other). Results from the multivariate Poisson regression model were expressed as adjusted incidence rate ratios. The risk of AVN over time following initiation of therapy was depicted by construction of a Kaplan–Meier curve. An alpha-level of 0·05 was used to determine statistical significance.

All analyses were conducted in SAS 8.2 (SAS Institute Inc., Cary, NC, USA) in accordance with STROBE guidelines (http://www.strobe-statement.org, accessed 15 June 2009).

## Results

As of July 2007 of a total of 4783 patients were enrolled in the ICGG Gaucher Registry; 3176 patients met the study inclusion criteria, i.e., received ERT, reported dates of therapy initiation and GD1 diagnosis, and reported age at diagnosis. The final analysis was based on 2700 patients; 476 of 3176 patients were excluded because they had a history of AVN before therapy initiation.

The incidence rate of new onset AVN among GD1 patients following initiation of therapy was 13·8 per 1000 person-years (Table I). Incidence rates then were stratified by interval in years between diagnosis and initiation of ERT. Among patients who initiated therapy within 2 years after diagnosis, the incidence rate of AVN following initiation of imiglucerase ranged from 8·0 to 8·2 per 1000 person-years. In contrast, the incidence rate of AVN among patients starting therapy more than 2 years after diagnosis (up to 30 years) rose progressively from 12·7 to 22·1 per 1000 person-years. Therefore, we consolidated our analysis of the rate of AVN on ERT into two groups: patients who started ERT within 2 years of diagnosis and those who started ERT 2 years or more after diagnosis.

**Table I:** Incidence rates of avascular necrosis according to time interval between Gaucher disease diagnosis and initiation of therapy.

	AVN cases	Number of patients	Person-years of follow-up	Incidence rate*
Total	213	2700	15 468	13·8
Years between Gaucher disease diagnosis and initiation of ERT with imiglucerase				
<1	24	639	3018	8·0
1–<2	17	408	2066	8·2
2–<5	30	394	2369	12·7
5–<10	35	375	2468	14·2
10–<20	48	440	2844	16·9
20–<30	33	244	1493	22·1
30+	26	200	1210	21·5

* Incidence rate per 1000 person-years.

Ascertainment of AVN was by MRI in 54% of cases and plain radiology for 41% of cases. The sites of AVN were the hip (52%), femur (21%), shoulder (6%), humerus (3%), tibia (2%), knee (2%), spine (<1%), ankle (<1%), wrist (<1%) and jaw (<1%); in 11% of cases, AVN occurred at multiple sites.

The characteristics of patients included in this analysis are depicted in Table II, stratified according to whether therapy was initiated within 2 years or 2 or more years after diagnosis. Additional patient characteristics are provided in Table SI. There were similar proportions of men (47%) and women (53%). The majority of patients were from the US (40%), Americas including Canada (21%) and Europe (25%). The most common race/ethnicities were non-Jewish Caucasians (41%) and Jewish (35%). Genotypes were reported for *c*. 70% of the patients; the majority (87%) had at least one N370S allele (26% homozygous, 61% heteroallelic).

**Table II:** Characteristics of patients.

	Years between Gaucher diagnosis and initiation of ERT with imiglucerase		
	<2 years (*N* = 1047)	≥2 years (*N* = 1653)	Total (*N* = 2700)
Gender, *n* (%)	*n* = 1047	*n* = 1653	*n* = 2700
Male	539 (51·5)	719 (43·5)	1258 (46·6)
Female	508 (48·5)	934 (56·5)	1442 (53·4)
Age at Gaucher diagnosis (years), *n* (%)	*n* = 1047	*n* = 1653	*n* = 2700
0–9	471 (45·0)	713 (43·1)	1184 (43·9)
10–19	171 (16·3)	317 (19·2)	488 (18·1)
20–29	120 (11·5)	278 (16·8)	398 (14·7)
30–39	111 (10·6)	151 (9·1)	262 (9·7)
40–49	84 (8·0)	107 (6·5)	191 (7·1)
50–59	46 (4·4)	47 (2·8)	93 (3·4)
≥60	44 (4·2)	40 (2·4)	84 (3·1)
Age at initiation of ERT with imiglucerase (years), *n* (%)	*n* = 1047	*n* = 1653	*n* = 2700
0–9	444 (42·4)	164 (9·9)	608 (22·5)
10–19	174 (16·6)	326 (19·7)	500 (18·5)
20–29	116 (11·1)	276 (16·7)	392 (14·5)
30–39	117 (11·2)	307 (18·6)	424 (15·7)
40–49	95 (9·1)	265 (16·0)	360 (13·3)
50–59	54 (5·2)	161 (9·7)	215 (8·0)
≥60	47 (4·5)	154 (9·3)	201 (7·4)
Year of Gaucher diagnosis, *n* (%)	*n* = 1047	*n* = 1653	*n* = 2700
Before 1991	21 (2·0)	1175 (71·1)	1196 (44·3)
1991–1999	504 (48·1)	387 (23·4)	891 (33·0)
2000 or later	522 (49·9)	91 (5·5)	613 (22·7)
Year of initiation of ERT with imiglucerase, *n* (%)	*n* = 1047	*n* = 1653	*n* = 2700
Before 1995	170 (16·2)	682 (41·3)	852 (31·6)
1995–1999	329 (31·4)	532 (32·2)	861 (31·9)
2000 or later	548 (52·3)	439 (26·6)	987 (36·6)
Genotype, *n* (%)	*n* = 704	*n* = 1166	*n* = 1870
N370S/N370S	169 (24·0)	317 (27·2)	486 (26·0)
N370S/Other	396 (56·3)	739 (63·4)	1135 (60·7)
Other/Other	139 (19·7)	110 (9·4)	249 (13·3)

In the group of patients who initiated therapy within 2 years following diagnosis, there were substantially more patients below age 10 years at therapy initiation (42%) and non-Jewish patients (76%) compared to the group of patients who initiated therapy 2 years or more following diagnosis (10% and 58%, respectively). However, the distributions of age at Gaucher diagnosis and genotypes were similar between the groups. As expected, 98% of patients who initiated therapy within 2 years following diagnosis were diagnosed after 1991 (when alglucerase first became available, i.e., during the ERT era), while the majority (71%) of patients in the group of patients who initiated therapy 2 years or more following diagnosis were diagnosed before 1991, i.e., during the pre-ERT era.

The haematological, visceral organ and skeletal characteristics at time of therapy initiation are shown in Table III. Overall, among patients with assessments reported, 43% had anaemia; 62% had platelet counts <120 × 10^9^/l; 89% had spleen volumes >5 multiples of normal, and 71% had liver volumes >1·25 multiples of normal. Bone pain around the time of first infusion was reported in 46% of all patients and low bone mineral density in 26%. There were no substantial differences between the <2 and ≥2 years groups in haematological and visceral findings or with regards to bone pain or bone mineral density. However, patients who initiated therapy 2 years or more following diagnosis were more likely to have reported a bone crisis around the time of initiation of treatment (16%, *n* = 123 of 772) compared to those in the <2 years group (6%, *n* = 35 of 607). Furthermore, there was a striking difference in prevalence of splenectomy in the two treatment groups: 32% (*n* = 522 of 1653) of the patients in the ≥2 years group had undergone splenectomy compared to only 6% (*n* = 65 of 1047) of the patients in whom treatment was initiated <2 years from date of diagnosis.

**Table III:** Clinical characteristics of patients.

	Years between Gaucher diagnosis and initiation of ERT with imiglucerase		
	<2 years (*N* = 1047)	≥2 years (*N*= 1653)	Total patients (*N* = 2700)
Anaemia at first infusion*†, *n* (%)	*N* = 598	*n* = 967	*n* = 1565
Yes	269 (45·0)	397 (41·1)	666 (42·6)
No	329 (55·0)	570 (58·9)	899 (57·4)
Platelet count (×10^9^/l) at first infusion*, *n* (%)	*N* = 600	*n* = 963	*n* = 1563
≥120	219 (36·5)	372 (38·6)	591 (37·8)
60–<120	290 (48·3)	378 (39·3)	668 (42·7)
<60	91 (15·2)	213 (22·1)	304 (19·4)
Spleen volume (multiples of normal) at first infusion‡, *n* (%)	*n* = 334	*n* = 435	*n* = 769
≤5	35 (10·5)	53 (12·2)	88 (11·4)
>5–≤15	161 (48·2)	185 (42·5)	346 (45·0)
>15	138 (41·3)	197 (45·3)	335 (43·6)
Liver volume (multiples of normal) at first infusion‡, *n* (%)	*n* = 317	*n* = 543	*n* = 860
≤1·25	90 (28·4)	163 (30·0)	253 (29·4)
>1·25–≤2·5	188 (59·3)	295 (54·3)	483 (56·2)
>2·5	39 (12·3)	85 (15·7)	124 (14·4)
Low bone mineral density at first infusion§¶, *n* (%)	*n* = 104	*n* = 137	*n* = 241
Yes	33 (31·7)	30 (21·9)	63 (26·1)
No	71 (68·3)	107 (78·1)	178 (73·9)
Bone pain at first infusion§, *n* (%)	*n* = 214	*n* = 487	*n* = 701
Yes	106 (49·5)	214 (43·9)	320 (45·6)
No	108 (50·5)	273 (56·1)	381 (54·4)
Bone crisis at first infusion§, *n* (%)	*n* = 607	*n* = 772	*n* = 1379
Yes	35 (5·8)	123 (15·9)	158 (11·5)
No	572 (94·2)	649 (84·1)	1221 (88·5)
Partial or total splenectomy prior to initiation of ERT with imiglucerase, *n* (%)	*n* = 1047	*n* = 1653	*n* = 2700
Yes	65 (6·2)	522 (31·6)	587 (21·7)
No	979 (93·5)	1127 (68·2)	2106 (78·0)
Unknown	3 (0·3)	4 (0·2)	7 (0·3)

* Represents the data point closest to the first infusion date, no more than 8 weeks before through to 2 weeks following the date of first infusion.

† Anaemia was defined according to age and gender norms for haemoglobin concentrations as follows: <120 g/l for males older than 12 years; <110 g/l for females older than 12 years; <105 g/l for children aged >2–12 years; <95 g/l for children aged 6 months–2 years; <101 g/l for children younger than 6 months of age.

‡ Represents the data point closest to the first infusion date, no more than 6 months before through to 6 weeks following the date of first infusion.

§ Represents the data point closest to the first infusion date, no more than 2 years before through to 6 weeks following the date of first infusion.

¶ Among patients less than age 50 years, lumbar spine *z*-score of −2 or lower; Among patients aged 50 years or older, lumbar spine *t*-score of −2·5 or lower.

The adjusted incidence rate ratio of AVN according to the time interval between GD diagnosis and initiation of therapy, which was analysed using a multivariate Poisson regression model, is shown in Table IV. Patients in whom ERT was initiated within 2 years of diagnosis of GD1 had approximately half the incidence rate of AVN (8·1 per 1000 person-years) compared to patients who started therapy 2 or more years after diagnosis (16·6 per 1000 person-years). The incidence rate difference between the two groups was 8·5 per 1000 person years [95% confidence interval (CI) 5·0–12·0 per 1000 person-years]. The multivariate Poisson regression analysis indicates that the adjusted incidence rate ratio of 0·59 (95% CI 0·36–0·96, *P* = 0·0343) represents a 41% decrease in the risk of AVN in patients who began ERT within 2 years following diagnosis. Splenectomy was an independent risk factor for AVN after adjusting for all other variables in the model. The adjusted incidence rate ratio for AVN among patients who were asplenic at the time of initiation of therapy was 2·23 (95% CI 1·61–3·08, *P* < 0·0001) compared to those who had an intact spleen. Women had a lower risk of AVN than men (adjusted incidence rate ratio 0·75, 95% CI 0·57–0·99, *P* = 0·0412). There were no other clear trends in the risk of AVN according to age at therapy initiation, age at Gaucher diagnosis, year of diagnosis, year of therapy initiation and genotype.

**Table IV:** Multivariate analysis: incidence rates and adjusted incidence rate ratios of AVN.

						95% confidence interval		
	AVN cases	Number of patients	Person-years of follow-up	Incidence rate*	Adjusted incidence rate ratio†	Lower	Upper	*P*-value
Total	213	2700	15 468	13·8				
Years between diagnosis and initiation of ERT with Imiglucerase								
<2	41	1047	5084	8·1	0·59	0·36	0·96	0·0343
≥2	172	1653	10 384	16·6	Ref			
Gender								
Female	110	1442	8499	12·9	0·75	0·57	0·99	0·0412
Male	103	1258	6969	14·8	Ref			
Age at Gaucher diagnosis								
0–9	97	1184	7421	13·1	1·23	0·66	2·3	0·5193
10–19	41	488	2563	16·0	1·30	0·71	2·36	0·3910
20–29	36	398	2223	16·2	1·19	0·65	2·19	0·5649
30–39	17	262	1415	12·0	Ref			
40–49	11	191	998	11·0	0·96	0·44	2·09	0·9100
50–59	7	93	491	14·3	1·03	0·40	2·65	0·9448
≥60	4	84	357	11·2	0·93	0·27	3·28	0·9156
Age at initiation of ERT with imiglucerase								
0–9	28	608	3547	7·9	0·67	0·35	1·28	0·2226
10–19	36	500	3025	11·9	0·84	0·50	1·41	0·5102
20–29	46	392	2228	20·6	1·42	0·90	2·25	0·1345
30–39	34	424	2342	14·5	Ref			
40–49	28	360	2123	13·2	0·93	0·55	1·58	0·8005
50–59	26	215	1218	21·3	1·63	0·93	2·88	0·0904
≥60	15	201	985	15·2	1·21	0·58	2·53	0·6049
Year of Gaucher diagnosis								
Before 1991	141	1196	8295	17·0	0·63	0·27	1·48	0·2880
1991–1999	56	891	5614	10·0	0·87	0·42	1·79	0·7072
2000 or Later	16	613	1559	10·3	Ref			
Year of initiation of ERT with imiglucerase								
Before 1995	117	852	6708	17·4	1·23	0·74	2·04	0·4242
1995–1999	60	861	5905	10·2	0·76	0·46	1·28	0·3059
2000 or Later	36	987	2855	12·6	Ref			
Splenectomy before initiation of ERT with imiglucerase‡								
Yes	94	587	3500	26·9	2·23	1·61	3·08	<0·0001
No	117	2106	11 943	9·8	Ref			
Genotype								
N370S/N370S	36	486	3109	11·6	Ref			
N370S/Other	103	1135	7415	13·9	1·11	0·74	1·67	0·6005
Other/Other	23	249	1612	14·3	1·21	0·68	2·13	0·5181
Not reported	51	830	3332	15·3	1·15	0·73	1·80	0·5402

* Incidence rate per 1000 person-years.

† Incidence rate ratios adjusted for all variables shown in the table through a multivariate Poisson regression model. Model excludes seven patients who received a splenectomy with an unknown date of procedure.

‡ Excludes seven patients who received a splenectomy but the date of the procedure was unknown.

Ref = reference category used to calculate adjusted incidence rate ratio for each variable.

**Fig 1:**
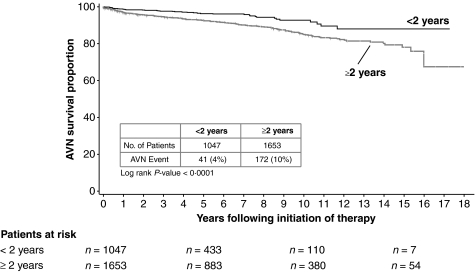
Kaplan–Meier curve.

The Kaplan–Meier analysis revealed a reduction in risk of AVN after 5 years of ERT among patients who began treatment within 2 years of diagnosis and this decrease in risk became more striking with subsequent follow up (Fig 1).

## Discussion

Avascular necrosis is a severe and irreversible complication of GD1 that frequently leads to functional disability (Wolfe & Taylor-Butler, 2000). Even after acute debilitating symptoms subside, patients suffer from chronic joint destruction, pain and progressive immobility. Joint replacement is frequently necessary, but, despite modern techniques, surgery is not always effective in restoring freedom from dependency on orthopaedic assistive devices or wheelchair confinement (Sims *et al*, 2008; Weinreb *et al*, 2007). Reports of patients on imiglucerase treatment, which is highly effective in reversing the haematological and splenic/hepatic manifestations of GD1, indicate decreased bone pain (El-Beshlawy *et al*, 2006) and bone crises (Charrow *et al*, 2007), improved marrow composition, bone mass (El-Beshlawy *et al*, 2006; Rosenthal *et al*, 1995), bone mineral density (Bembi *et al*, 2002; Wenstrup *et al*, 2007), growth rates in children (Bembi *et al*, 2002; El-Beshlawy *et al*, 2006) and decreased numbers of other skeletal complications (Sims *et al*, 2008).

Using data from the ICGG Gaucher Registry, this study reports the incidence rates of AVN in a large, international, diverse GD1 patient population after initiation of ERT. The overall risk of AVN following treatment initiation was 13·8 per 1000 person years of follow-up. The risk of AVN on ERT was lower among patients who began treatment within 2 years of diagnosis compared to those who started treatment after 2 years or more. The risk following treatment initiation was not associated with any *GBA* genotype category but was highly associated with splenectomy status.

The natural history and clinical manifestations of GD1 are markedly heterogeneous. In particular, the occurrence of AVN is unpredictable and its predisposing risk factors and mechanistic basis are not yet understood. There is no apparent correlation of risk of AVN with the severity of splenic, hepatic or haematological disease. However, we observed that patients who started treatment 2 years or more after GD diagnosis reported more bone crises around the time of treatment initiation (Table III). It is possible that bone crises may be an intermediate step in disease progression and indicator of future development of AVN. In addition, one report (Wolfe & Taylor-Butler, 2000) linked AVN and bone disease in GD1 with blood cell damage, which may be associated with activation of the coagulation cascade including the abnormal presence of thrombin-anti-thrombin complexes or increases in D-dimer (a particular fibrin degradation product) concentrations (Hollak *et al*, 1997). Abnormalities in red cell deformability may also be associated with GD (Bax *et al*, 2005) and AVN (Wolfe & Taylor-Butler, 2000). Persistent increases in plasma macrophage inflammatory proteins MIP-α and MIP-β as well as other biomarkers of chronic inflammation have also been reported in patients both with and without GD1-associated AVN (van Breemen *et al*, 2007). Perturbations in immunoregulatory anti-inflammatory cytokines such as interleukin-10 have also been reported in association with AVN (Mori *et al*, 2001) as well as in experimental GD models and GD1 patients (de Fost *et al*, 2008; Kacher & Futerman, 2009).

It is unclear as to how these abnormalities relate to the primary pathophysiology of GD1 or whether they directly contribute to the development of AVN. Patients who were diagnosed with GD1 before ERT was available often suffered from progressive disease complications. Currently, however, once diagnosed, symptomatic patients may be promptly treated. The incremental risk for AVN with increasing interval from diagnosis to initiation of ERT suggests that the probability of developing post-treatment AVN is substantially impacted by the total duration of the pre-treatment phase. Studies using animal models (Enquist *et al*, 2006) may provide insight into whether prolonged untreated GD1 leads to refractory changes in bone microvasculature due to chronic inflammation that may limit the effectiveness of ERT for prevention of AVN.

For patients without reported osteonecrosis who begin treatment 2 or more years after diagnosis of GD1, the Kaplan–Meier analysis suggests there is a 20% probability of developing AVN within 15–16 years (Fig 1). In children and young adults, this represents a significant lifetime risk. However, the multivariate Poisson regression model shows that, aside from timing of therapy initiation, prior splenectomy is also an independent risk factor for the development of AVN while on imiglucerase therapy. Because nearly one third of the patients who began treatment 2 or more years after diagnosis of GD1 had undergone splenectomy and as this procedure is performed much more rarely at present, our projection of lifetime risk of post-ERT AVN may be overstated for newly diagnosed patients.

The role of splenectomy in increasing the risk of AVN has been controversial with reports both in favour (Fleshner *et al*, 1991) as well as against such an association (Lee, 1982). Here, we found the risk of AVN in splenectomized patients after starting ERT is more than twofold higher than that of non-splenectomized patients; furthermore this increased risk persisted with enzyme therapy. Prior to the availability of ERT, splenectomy was commonly performed to control signs and symptoms of GD1 including severe cytopenias and abdominal discomfort (Cox *et al*, 2008). Splenectomy should only be considered in exceptional circumstances after assessment by a physician experienced in the management of GD (Cox *et al*, 2008).

This analysis has a number of limitations related to the Gaucher Registry design and study exclusion criteria. All Registry data are retrospective and unaudited. We have not attempted to ascertain the incidence of AVN in asymptomatic or mildly affected GD1 patients who are not considered to be candidates for ERT. Because criteria for initiation of ERT are not dictated by the Registry and vary considerably among physicians and geographic locales, the population of treated patients that analysed in this study was clinically heterogeneous. The treatment groups were comparable in terms of ERT dosing. However, we did not attempt to determine whether imiglucerase dose or schedule affects the overall incidence of AVN.

Furthermore, because this was not a randomized trial, there is always the concern of residual confounding, particularly according to variables not captured in the Registry. We were unable to assess the influence of known non-GD1 related risk factors for AVN, such as corticosteroids, alcohol, trauma and cancer (Wolfe & Taylor-Butler, 2000). However, we believe residual confounding according to these variables is unlikely, as bias would have been introduced into our study only if the distributions of such risk factors varied according to the time interval between Gaucher diagnosis and initiation of therapy.

A common concern in any observational study that relies on diagnostic screening for outcome ascertainment is the possibility of lead-time bias (Rothman, 2002). If this bias were present in our study, the differences in the incidence of AVN may be explained by systematically different levels of AVN screening in the study groups. It may be hypothesized that patients who initiated therapy sooner following Gaucher diagnosis may have received more screening and follow-up than others. This increased screening and follow-up would be expected to detect onset of AVN earlier and more frequently. However, our data indicate lower rates of AVN in patients who initiated therapy within 2 years following diagnosis, suggesting that lead-time bias could not have played a role in our study.

In this study we did not assess the incidence rates of AVN in untreated patients as such an analysis could invite assumptions about the effect of ERT on rates of AVN in Gaucher disease. The analytical methods used in this paper are not designed to assess the therapeutic effect of any particular treatment. A separate study of the factors that influence the rates of AVN in untreated Gaucher disease is in progress.

The Registry does not have standardized diagnostic criteria for reporting AVN. AVN may, at times, be asymptomatic and evolve slowly over years rendering the diagnosis difficult. The Registry has formulated a recommended schedule of skeletal assessments but compliance is voluntary. The requested anatomical sites for serial imaging are restricted to the hips, femora, pelvis, and, more recently, the lumbosacral spine. Asymptomatic AVN sites in other skeletal areas may therefore be missed, causing underestimation of the incidence in all groups that we studied. Moreover, we do not yet know the extent to which a radiological finding of AVN in an asymptomatic patient correlates with eventual functional disability and decreased health-related quality of life. Therefore, some caution should be exercised in concluding that the decreased incidence of *de novo* post-treatment AVN that we observed in GD1 patients in whom imiglucerase infusions were initiated within 2 years of diagnosis translates into an unequivocal clinical benefit. However, our findings clearly demonstrate that in some patients, later initiation of therapy following diagnosis can potentially result in skeletal pathology that may cause irreversible morbidity and disability. Pending a better understanding of the biological mechanisms that contribute to the development of AVN in patients with GD1 our findings support earlier rather than later therapeutic intervention in symptomatic patients.

## Supporting information

**Table SI.** Additional characteristics of patients.
