# Supplementary material for: Timing of initiation of enzyme replacement therapy after diagnosis of type 1 Gaucher disease: effect on incidence of avascular necrosis
Source: Br J Haematol. 2009 Nov;147(4):561–70. doi: 10.1111/j.1365-2141.2009.07872.x (PMC2774157; doi:10.1111/j.1365-2141.2009.07872.x)
Supplement: Supplementary file 1 [file bjh0147-0561-SD1.doc]

**Supplementary Data**

**Table 1S. Additional Characteristics of Patients**

|  | **Years Between Gaucher Diagnosis  and Initiation of ERT with Imiglucerase** | |  |
| --- | --- | --- | --- |
|  | **<2 Years  (N=1047)** | **>2 Years  (N=1653)** | **Total  (N=2700)** |
| **Ethnicity, n (%)** | **n=870** | **n=1137** | **n=2007** |
| Caucasian, Non-Jewish | 379 ( 43.6) | 450 ( 39.6) | 829 ( 41.3) |
| Jewish | 212 ( 24.4) | 483 ( 42.5) | 695 ( 34.6) |
| African-American/Caribbean | 104 ( 12.0) | 89 ( 7.8) | 193 ( 9.6) |
| Hispanic | 79 ( 9.1) | 59 ( 5.2) | 138 ( 6.9) |
| Arab | 54 ( 6.2) | 16 ( 1.4) | 70 ( 3.5) |
| Asian | 22 ( 2.5) | 16 ( 1.4) | 38 ( 1.9) |
| Multi-Ethnic, Non Jewish or Arab | 11 ( 1.3) | 19 ( 1.7) | 30 ( 1.5) |
| American Indian | 9 ( 1.0) | 5 ( 0.4) | 14 ( 0.7) |
| **Geographic Region, n (%)** | **n=1047** | **n=1653** | **n=2700** |
| Americas**^†^** | 287 ( 27.4) | 273 ( 16.5) | 560 ( 20.7) |
| Europe | 238 ( 22.7) | 446 ( 27.0) | 684 ( 25.3) |
| Middle East^‡^ | 84 ( 8.0) | 170 ( 10.3) | 254 ( 9.4) |
| Asia, Pacific, S. Africa | 39 ( 3.7) | 71 ( 4.3) | 110 ( 4.1) |
| USA | 399 ( 38.1) | 693 ( 41.9) | 1092 ( 40.4) |
| **Average Dose of ERT with Imiglucerase (u/kg/2wks)*, n (%)** | **n=1037** | **n=1620** | **n=2657** |
| < 15 | 65 ( 6.3) | 231 ( 14.3) | 296 ( 11.1) |
| 15 to < 30 | 254 ( 24.5) | 450 ( 27.8) | 704 ( 26.5) |
| 30 to < 45 | 255 ( 24.6) | 448 ( 27.7) | 703 ( 26.5) |
| 45 to < 60 | 265 ( 25.6) | 255 ( 15.7) | 520 ( 19.6) |
| >=60 | 198 ( 19.1) | 236 ( 14.6) | 434 ( 16.3) |

**^†^**Americas include Argentina, Bolivia, Brazil, Canada, Chile, Colombia, Costa Rica, Ecuador, Guatemala, Mexico, Panama, Paraguay, Peru, Uruguay and Venezuela.

^‡^Middle East includes Israel, Egypt, Oman, Saudi Arabia.

*Average dose within 3 years following initiation of ERT with imiglucerase.
